# The effect of a ketogenic diet versus a high-carbohydrate, low-fat diet on sleep, cognition, thyroid function, and cardiovascular health independent of weight loss: study protocol for a randomized controlled trial

**DOI:** 10.1186/s13063-018-2462-5

**Published:** 2018-01-23

**Authors:** Stella Iacovides, Rebecca M. Meiring

**Affiliations:** 1 Brain Function Research Group, School of Physiology, Faculty of Health Sciences, University of the Witwatersrand, 7 York Rd, Parktown, Johannesburg, South Africa; 2 Movement Physiology Research Laboratory, School of Physiology, Faculty of Health Sciences, University of the Witwatersrand, 7 York Rd, Parktown, Johannesburg, South Africa

**Keywords:** High-fat, Low-carbohydrate, Ketogenic diet, Metabolism, Cognition, Thyroid function, Sleep, Healthy participants

## Abstract

**Background:**

Many physiological health benefits observed after following a ketogenic diet (KD) can be attributed to the associated weight loss. The KD has become more prominent as a popular health choice, not only in obese/overweight individuals, but also in healthy adults. The study aims to determine the effects of a KD, independent of weight loss, on various aspects of physiological health including: sleep, thyroid function, cognition, and cardio-metabolic health. The study will also aim to determine whether a change in basal metabolic rate may be associated with any changes observed.

**Methods:**

Twenty healthy men and women between 18 and 50 years of age will take part in this study. In a randomized controlled, cross-over design, participants will follow two isocaloric diets: a high-carbohydrate, low-fat diet (55% CHO, 20% fat, 25% protein) and a KD (15% CHO, 60% fat, 25% protein). Each dietary intervention will last for a minimum of 3 weeks, with a 1-week washout period in between. Before and after each diet, participants will be assessed for sleep quality, cognitive function, thyroid function, and basal metabolic rate. A blood sample will also be taken for the measurement of cardio-metabolic and immune markers.

**Discussion:**

The present study will help in understanding the potential effects of a KD on aspects of physiological health in healthy adults, without the confounding factor of weight loss. The study aims to fill a significant void in the academic literature with regards to the benefits and/or risks of a KD in a healthy population, but will also explore whether diet-related metabolic changes may be responsible for the changes observed in physiological health.

**Trial registration:**

Pan African Clinical Trial Registry (www.pactr.org), trial number: PACTR201707002406306. Registered on 20 July 2017.

**Electronic supplementary material:**

The online version of this article (10.1186/s13063-018-2462-5) contains supplementary material, which is available to authorized users.

## Background

Obesity, both independently, and in association with other obesity-related diseases, leads to adverse physiological effects of many aspects of health including sleep [1, 2], cognitive function [3, 4], and cardiovascular health [5]. A reduction in body fat is a significant treatment target used to improve obesity-induced adverse effects on each of these physiological health variables [6–8]. However, studies investigating the effect of diet on the physiological aspects of health independent of the loss of weight (whether total mass or fat mass) are lacking. Diets low in carbohydrate and high in fat, or ketogenic diets (KD), are conventionally used to treat epilepsy and neurodegenerative disorders [9, 10], but have been relatively popular for weight loss since the 1800s [11]. Studies on KDs vary in their daily limit of carbohydrate intake; carbohydrate consumption may be restricted to as low as 4% [12, 13] up to 40% [14, 15] of daily caloric intake. The fundamental principle of the KD, however, is severe restriction of dietary carbohydrate consumption, with a concurrent increase in dietary fat to compensate for the energy deficit, resulting in the promotion of lipid oxidation to produce ketones as an energy source (as opposed to glucose), and thus, a metabolic state of nutritional ketosis [16].

Obesity affects various aspects of physiological health, including sleep and cognition. Compared to controls, obese individuals have more night-time wakefulness and less total sleep time [17]. Furthermore obesity with [18] and without [17] sleep apnea is associated with fatigue, daytime sleepiness, and poor sleep quality. A reduction in body fat improves obesity-related adverse effects on sleep architecture and daytime sleepiness [7]. In populations with sleep abnormalities, a KD has been shown to reduce sleepiness [19, 20] and normalize sleep architecture [19, 21] but this change was in association with a loss of body mass, particularly body fat [19]. In healthy, non-obese men who were good sleepers, a KD diet increases slow-wave sleep and decreases rapid-eye-movement sleep [22] compared to a high-carbohydrate, low-fat (HCLF) diet [1]. Studies investigating the effect of the KD on sleep, however, are limited by their study populations and small sample sizes.

Obesity is also a risk factor for dementia and adverse changes to brain structure and function [3]. The relationship between obesity and cognitive decline is likely to be bidirectional, with suggestions that a gain in body fat may, at least in part, be the result of a neurological predisposition characterized by reduced executive brain function, but that obesity further compounds the adverse effects on the brain, possibly via mechanisms involving inflammatory pathways, elevated lipids and/or insulin resistance [8]. There is some evidence to suggest that loss of body mass has the potential to reverse some of the negative obesity-related effects on cognition, particularly with regards to working memory following significant weight loss [12]. Independent of obesity, prospective human studies have identified high-fat diets, particularly diets rich in saturated fats, as risk factors for developing Alzheimer’s disease and dementia [23–26]. Most human studies highlight that specific types of fats are more important than total fat intake; in particular, omega-6 and saturated fatty acids are associated with reduced cognitive performance [24, 27–29]. Randomized controlled trials (RCTs) investigating the effects of the KD on cognition in younger, healthy individuals, are lacking in the academic literature. Therefore, it is yet to be determined whether increased adiposity, or the consumption of high-fat diets, is responsible for cognitive decline.

Dietary effects on cardiovascular function have been investigated more than any other physiological function. However, the majority of studies have been conducted in obese and/or overweight individuals, who are likely to be in an obesity-induced, low-grade pro-inflammatory state. Apprehensions exist that the KD, with its concomitant high intake of total and saturated fat, adversely affects blood lipid levels and hence, increases the risk for cardiovascular disease (CVD) [30–36]. Traditionally, a low-fat diet is prescribed for body fat reduction for obese patients with or at risk for CVD and it is suggested that the fat loss following any diet restriction and/or exercise accounts for the favorable improvements seen in cardiovascular health [37, 38]. However, a meta-analysis on 23 controlled trials involving 1141 obese patients, reported that the KD diet in fact has favorable effects on cardiovascular health [39]. In particular, the KD is associated with significant reductions in plasma triglycerides, fasting plasma glucose, glycated hemoglobin, plasma insulin, C-reactive protein, systolic and diastolic blood pressures, total body mass, abdominal circumference, as well as significant increases in high-density lipoprotein (HDL) cholesterol, with no significant changes in low-density lipoprotein (LDL) cholesterol [39].

The inconsistencies in the results of studies comparing physiological variables between the KD with the current recommended HCLF diet, are further confounded by the fact that total body mass loss is associated with both these diets, particularly with calorie restriction. Given that physiological factors, such as sleep and cardiovascular health, are altered by a reduction in body fat, especially in overweight and obese populations, it is difficult to differentiate between the effects of the loss of fat mass or dietary composition. It is, therefore, important to explore the physiological outcomes of these diets in the absence of any change in body mass.

Further, it is important to note that even with an increase in calorie intake on a KD, a greater loss of body mass has been reported in overweight adolescents compared with those on the HCLF diet, and it has been suggested that a non-calorie restricted KD may be more effective than a very-low-calorie diet due a greater maintenance of metabolic rate, which is a consequence of a higher caloric intake [40]. Despite the most common application for the KD being a reduction in total body mass, there is astonishingly little research investigating the effects of a KD, compared with the conventional HCLF diet, on basal metabolic rate (BMR) or thyroid function [41, 42]. Changes in the thyroid hormones triiodothyronine (T3) and thyroxine (T4) that contribute to maintenance of metabolic rate due to a low-carbohydrate diet have produced mixed results [41–43]. Importantly, however, caloric intake in these studies was not controlled. Instead, greater loss of body mass on the KD compared with HCLF diets, in obese and/or overweight individuals, has been attributed to spontaneous reduction in caloric intake [41, 44, 45], reduced hunger and/or increased satiety due to increased fat consumption [46–49], or an inhibitory effect of β-hydroxybutyrate (BOHB) (the major circulating ketone body) on appetite [50]. Given the persistent metabolic adaptation that follows loss of total body mass and/or calorie restriction, namely adaptive thermogenesis, which promotes an overall decline in energy expenditure and a reduced BMR [51, 52], it is intriguing to determine whether diet composition, independent of a loss of body mass, caloric intake, and levels of physical activity, has any significant metabolic effects.

A controlled, cross-over dietary intervention in healthy individuals, incorporating vital aspects of physiological health into one study and negating the effect of body mass loss and calorie intake, has not yet been done. Given that the majority of studies investigating the KD have: (1) been done in overweight/obese populations, with or without diabetes mellitus and/or insulin resistance, (2) did not control for total caloric intake and levels of physical activity, and (3) did not incorporate a cross-over of subjects doing both diets, this study will account for the many study limitations that exist in the current academic literature by controlling for body mass, existing disease, and calorie intake. Therefore, we wish to determine, in a randomized-controlled cross-over study design, whether a KD, compared with a HCLF diet, affects sleep, cognition, thyroid function, and cardiovascular effects in a generally healthy, non-obese/overweight population while maintaining body mass on the two diets. In addition, we aim to determine whether BMR is different between the ketogenic and HCLF diets in normal healthy adults and whether this difference is associated with any difference in sleep, cognitive and cardiovascular physiology.

## Methods

### Study design

**Fig. 1:**
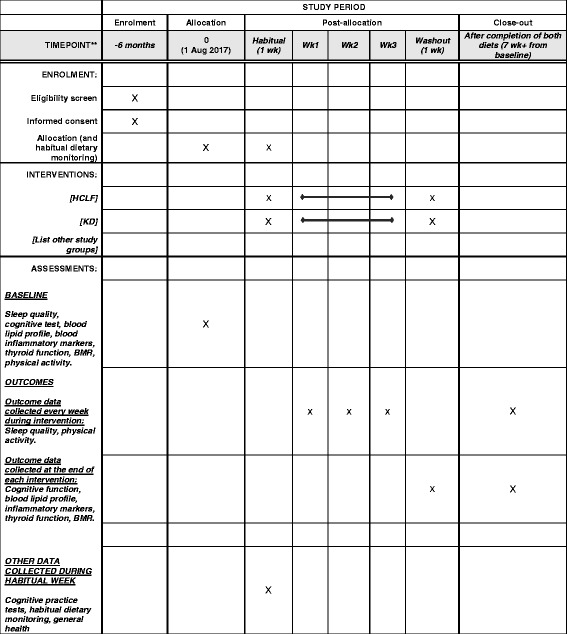
Standard Protocol Items: Recommendations for Interventional Trials (SPIRIT) schedule of enrollment, interventions, and assessments for the duration of the study. HCLF = High carbohydrate, low fat diet; KD= ketogenic diet, BMR = basal metabolic rate

**Fig. 2:**
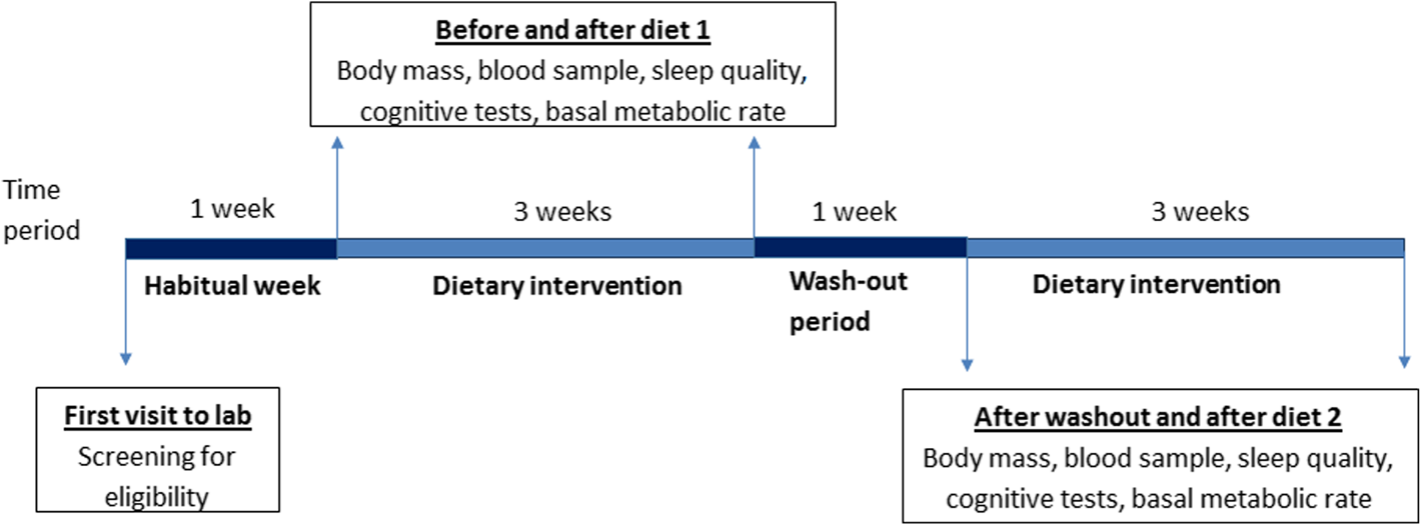
Timeline and details of study procedures

The design of the study is a randomized controlled, cross-over trial. The study will follow the Standard Protocol Items: Recommendations for Interventional Trials (SPIRIT) guidelines for the design and conduct of a trial (Fig. 1; Additional file 1) [53]. As outlined in Fig. 2, participants will be asked to visit the Movement Physiology Research Laboratory on five occasions; the first visit will be for screening the volunteers to ensure that they fit the study criteria. Thereafter, eligible participants will return to the laboratory before the start of, and after, each dietary intervention. Following 1 week of normal habitual dietary monitoring, each participant will follow two isocaloric diets (a KD and a high-carbohydrate, low-fat (HCLF) diet) in a randomized order and separated by a 1-week washout period. Randomization of the order of the diets will be done using the Microsoft Excel 2010 (Version 14.0) CHOOSE and RANDBETWEEN functions. These functions (=CHOOSE(RANDBETWEEN(1,2), “A”,“B”) are able to assign each new participant into one of the two diets. Each dietary intervention will last for a minimum period of 3 weeks. In the KD it is critical for the participant to remain in a ketogenic state for three consecutive weeks. Should the participant leave the ketogenic state (as assessed by blood ketone levels), the dietary intervention period will be prolonged until the consecutive, 3-week ketogenic state is achieved. If a participant does not reach a ketogenic state in 2 weeks, we will exclude them, as it is indicative of non-compliance. However, we will be more lenient with someone who has entered a ketogenic state but may struggle to maintain the state, in which case we will guide them by closely monitoring their diet to ensure that their carbohydrate intake is suitably low and that their fat intake is suitably high. We will also look at the content of each participant’s diet to ensure that the foods do not contain imperceptible carbohydrates or that their protein content is not too high, which may result in gluconeogenesis. We will continuously encourage pure fat intake, e.g., cooking with olive oil, butter or coconut oil. Should a participant leave the ketogenic state more than five times they will be excluded from the study.

The principal investigator (PI) who is collecting the outcome measures will not be blinded to the diet allocation because feedback may need to be provided on a daily basis, to encourage dietary compliance. A researcher that will conduct the statistical analysis will, however, be blinded to the order in which the diets were undertaken.

### Participants

Twenty healthy individuals, between the ages of 18 and 50 years, will be asked to volunteer in this study. A total of 20 participants would provide 85% power to obtain a medium effect size for BMR (Cohen’s *d* effect size = 0.3) [54]. Using standardized and customized screening questionnaires for sleep quality and general health, volunteers will be screened to ensure that they are free from any chronic illness, depression, sleep disorders, for at least 6 months prior to the start of the study. Volunteers will be excluded if they are overweight or obese (BMI ≥ 26 kg/m^2^), have any adverse cardiac or metabolic conditions such as type 1 or 2 diabetes mellitus, hypercholesterolemia or hypertension confirmed by a clinician. The Pittsburgh Sleep Quality Index (PSQI) [55] and the General Health Questionnaire (GHQ) [56] will be used to assess quality of sleep and psychological health, respectively. Eligible participants will be asked to maintain the same level of physical activity throughout the study, which will be monitored using the standardized Global Physical Activity Questionnaire (GPAQ) [57]. If participants are taking a daily vitamin/mineral supplement, they will be asked to continue taking it as usual. If they are not taking any supplement they will be asked not to start taking one during the study. Using the dietary data obtained from 1 week of habitual dietary monitoring at the start of the study, daily calorie intake will be calculated for each participant and the total amount of calories consumed daily during both dietary interventions will be based on this value.

### Dietary intervention

Participants will, in a random order, follow a KD (15% carbohydrate, 60% fat, and 25% protein) and a HCLF diet (55% carbohydrate, 20% fat, and 25% protein). Each dietary intervention will last for a minimum of 3 weeks, with a 1-week washout period in between each diet, during which time participants will be asked to follow their habitual diet (as during the first week of the study). The PI will provide each participant with meal plans that are specific to each dietary intervention, but will maintain an identical calorie intake to that which was consumed, on average, over the first week of following each participant’s habitual diet. Therefore, the PI will formulate individualized meal plans with the correct respective macronutrient content for each dietary intervention according to each participant’s habitual calorie intake. The PI will also educate each participant on the fundamentals of each dietary intervention and provide foodlists, and other resources containing information on dietary macronutrient compositions and calories, to assist each participant to make food choices of their preference, but in line with the dietary intervention. The PI will also be easily assessable to each participant for guidance and/or advice, as well as motivation, throughout the study. Ultimately, these measures should assist with participant compliance.

### Dietary monitoring

Throughout the study (i.e., during the first week (habitual week), during both dietary interventions and during the washout period), participants will be asked to record a detailed account of their daily dietary consumption using specialized, but commercially available, calorie-counter applications (MyFitnessPal, version 7, Under Armour). The applications have the functionality to export detailed diet data for later analysis. In addition, the PI will be able to remotely monitor (smart devices will be connected), and critically analyze and assess, macronutrient composition and calorie intake, in order to provide each participant with daily feedback during the diets. The feedback provided will either be positive, encouraging comments, or advice on how to best amend their diet for the purpose of the study. In order to ensure that a nutritional ketosis is maintained for three consecutive weeks of being on the KD, blood levels of β-hydroxybutyrate (BOHB) will be determined using a finger-prick test and a β-ketone handheld analyzser (Freestyle Optium, Abbott Diabetes Care Ltd., United Kingdom). Levels of BOHB will be measured before and after each dietary intervention, as well as at the end of each week while participants are on the KD. During the intervention, each participant will be provided with a β-ketone handheld analyzer and shown explicitly how to use the device. Participants will then be able to monitor ketones at home themselves without having to return each week for laboratory assessment. Participants will be required to take a photo of the value recorded by the handheld analyzer and send that photo to the PI. Monitoring of BOHB levels will be done to confirm the ketogenic state in each participant, and once achieved, the BOHB assessment will be repeated every second day to ensure that the ketogenic state is maintained for three consecutive weeks. Maintenance of a ketogenic state for three consecutive weeks will be a critical requirement of the study. When participants leave the ketogenic state (considered as BOHB levels below 0.4 nM), the dietary intervention period will be prolonged until the consecutive 3-week ketogenic state is achieved.

### Assessments

#### Home assessments

During each dietary intervention, in addition to daily dietary recall, participants will be provided with study-specific morning and evening questionnaires to assess daily subjective measures of sleep, mood, and exercise activity. Further, at the end of each week, physical activity will be assessed [57].

##### Sleep, mood, and exercise questionnaires

For the duration of the study period, participants will be required to complete a customized sleep diary every morning (morning questionnaire) to assess perceived sleep quality as well as morning vigilance on two separate 100-mm Visual Analogue Scales (VAS), anchored from the “worst sleep” to the “best sleep ever,” and “not at all fresh and alert” to the “most fresh and alert ever,” respectively. The morning questionnaire will also provide information on each participant’s sleeping habits, such as what time they went to bed, how long they think it took them to fall asleep, and what time they woke up. Thus, information on each participant’s sleep-wake cycles can be retrieved. Each evening, throughout the study, participants will be required to complete a customized evening questionnaire to assess current mood on a 100-mm VAS anchored from “worst mood ever” to “best mood ever.” The evening questionnaire also asks for information on the type, duration, and intensity of any exercise performed during the day. At the end of each week, physical activity will be further assessed using the standardized GPAQ [57].

#### Laboratory assessments

Before and after each dietary intervention (i.e., four times), participants will return to the laboratory after an overnight fast, for anthropometric measurements and to provide a blood sample. Tests to assess cognitive function tests and BMR will also be assessed at each of these visits, and validated and standardized Pittsburgh Sleep Quality Index (PSQI) [55] questionnaires will be administered to assess sleep quality.

##### Anthropometry

Height (to the nearest centimeter) and body mass (to the nearest 100 g) will be measured using a stadiometer (Holtain Ltd., Crymych, UK) and electronic scale (Dismed, Miami, FL, USA) respectively. Participants will be measured without shoes and while wearing light clothing. Biceps, triceps, supra-iliac and sub-scapularis skinfolds (to the nearest 2 mm) using Holtain skinfold calipers (Holtain Ltd., Crymych UK), will be used to determine body fat percentage of each participant [58].

##### Cognitive function

Cognitive function will be assessed using a battery of computerized cognitive tests (Cogstate computerized tests (www.cogstate.com)) performed on a laptop computer with headphones. In order to avoid a learning effect of performing these cognitive function tests, participants will be required to perform the battery of tests and repeat it three times during the habitual diet screening week. Cognitive tests will include: (1) Groton Maze learning task (to assess executive function), (2) Identification test (to assess attention), (3) Psychomotor vigilance task (to assess reaction times), (4) One-card learning task (to assess visual learning), and (5) Two-back test (to assess working memory). This battery of tests will be done during the habitual dietary screening week, as well as before and after each dietary intervention.

##### Blood sampling and collection

After an overnight fast (minimum of 10 h) a 10-ml venous blood sample will be drawn by venous puncture from the brachial vein of the participant for the assessment of biomarkers of cardiovascular and metabolic health. These biomarkers include lipid profiles (concentrations of triglycerides, LDL, HDL and total cholesterol), interleukin-6 (IL-6), interleukin-1 (IL-1), high-sensitivity C-reactive protein (hsCRP), tumor necrosis factor alpha (TNF-α), ferritin, and serum amyloid P. The blood sample will also be used to assess thyroid function by measuring levels of thyroid stimulating hormone (TSH), T3 and T4. Blood samples will be taken before and after both dietary interventions. Samples will be analyzed by a reputable external laboratory (Clinical Laboratory Service, Johannesburg, South Africa).

##### Basal metabolic rate

Basal metabolic rate will be measured using respiratory gas analysis via a computerized metabolic system (Quark ergo, COSMED, Rome, Italy). Participants will lie supine on a plinth for a period of 15 min with a face mask, covering their mouth and nose, connected to the metabolic system. Oxygen consumption, carbon dioxide production, ventilation, and respiratory rate will be recorded throughout the procedure. Average energy expenditure (kcal/min/kg) over the last five of the 15 min will then be recorded as BMR. The room temperature of the laboratory will be maintained at 27 °C for all participants.

### Outcomes

All outcomes will be measured at baseline, and before and after 3 weeks of each dietary intervention. The primary outcome will be diet-induced change in sleep quality, cognitive function, markers of inflammation and cardiovascular health status. The secondary outcomes will be a change in BMR and thyroid function.

### Data analysis

Data analysis will be done using IBM SPSS Statistics (version 24, IBM Corporation, NY, USA). A two-tailed statistical significance will be accepted at *P* < 0.05. Each outcome variable assessed will be analyzed according to diet (KD or HCLF) and time (before and after each diet) using a repeated-measures two-way analysis of variance (ANOVA). Where appropriate, a Student-Newman-Keuls post-hoc test will be used to assess the origin of any significant differences detected by the ANOVA models. Variables will be adjusted for potential confounders which will be determined using multiple linear regressions. A power analysis has indicated that total of 20 participants would provide 85% power to obtain a medium effect size for BMR (Cohen’s *d* effect size = 0.3) [54].

## Discussion

There is little understanding of how dietary macronutrient composition affects various physiological measures of health in a healthy population. Our study aims to investigate the effect of a KD, independent of weight loss, on the physiological outcomes of sleep, cognitive function, thyroid function, metabolic rate, and cardiovascular health, including inflammatory markers, in a non-overweight/non-obese population. Due to the confounding effects of obesity and chronic illness on physiological health, we believe that negating the effect of weight loss, chronic disease, and overweight/obesity, we can add valuable insight into the direct effects of macronutrient composition (diet) on various aspects of physiological health. The increasing popularity of a KD in the general population warrants investigation into the effects of this diet on other physiological aspects of health including sleep, cognitive and thyroid function, and cardiovascular health. Limitations in existing study designs include that most studies compare different groups of individuals on different diets (i.e., not a cross-over design), participants included in these studies are those who are obese/overweight who likely also have underlying chronic illnesses like insulin resistance, metabolic syndrome or dyslipidemia, and importantly, most studies do not control for calorie-intake (i.e., dietary interventions are not isocaloric) nor physical activity (calorie output). The present study design aims to control for all the above limitations and to determine the effects of diet on various physiological parameters in the absence of a variation in calorie input and output, and thus, the absence of body mass change. Another limitation of studies is that the level of carbohydrate restriction in the KD varies from study to study. We chose a level of 15% of calorie derivation from carbohydrates for our KD based on more recent RCTs that have restricted their diets to 20% carbohydrates [59, 60].

Weight loss is an outcome measure of most studies; however, mechanisms to explain potential changes in weight are poorly explored and the observed weight loss is often used to explain other effects on physiological health [6–8]. Despite a large number of studies investigating the effects of diet on weight loss, the effect of diet on thyroid function and BMR is poorly understood. Following short-term and sustained weight loss, adaptive thermogenesis occurs, where total energy expenditure, non-resting energy expenditure and resting energy expenditure, are all significantly reduced compared with pre-weight-loss measures [52]. Thus, the decline in overall energy expenditure, favors the regain of the lost weight, and this metabolic compensation persists well beyond the period of active weight loss [38]. BMR is the largest contributor of total daily energy expenditure but is known to be the least modifiable component [61]. Exercise-associated thermogenesis (EAT), non-exercise-associate thermogenesis (NEAT), and TEF (the thermic effect of food) constitute the three components of the more changeable components of metabolism, and are known collectively as NREE (non-resting energy expenditure). Given that NEAT (i.e., non-structured exercise, including walking, standing, fidgeting, doing housework, etc.) is the largest component of the changeable aspects of metabolism, and given that metabolic compensation following weight loss largely targets NEAT, but may also modify your motivation to exercise (EAT), in this study we wish to reduce these confounders by (1) monitoring physical activity levels, but also, more importantly, (2) implementing a dietary intervention without a calorie deficit. In particular, each dietary intervention (macronutrient composition) will be designed according to each participant’s habitual daily calorie consumption. The purpose is to mitigate a change in body mass and, therefore, the associated changes in metabolism, in order to determine whether dietary macronutrient composition alone (i.e., changes in the relative contribution of each macronutrient consumed daily, without changing total calorie intake) can affect metabolic rate, and related thyroid function.

Furthermore, our study aims to investigate inflammatory markers that are risk factors for the development of CVD. A limited number of studies investigating the effects of diet on CRP and adipocytokines, such as TNF-α, have been performed and have produced conflicting results [15, 62–64]. It is also important to note that their results are further confounded by the populations studied, given that both CRP and adipocytokines are affected by obesity and weight loss [65, 66]. We therefore believe that these measurements will add valuable insight to cardiovascular function and inflammatory markers in a healthy population.

As the popularity of following a KD increases and as people begin to question the relevance of the high carbohydrate diet in contributing positively to the maintenance of one’s metabolic and physiological health, the present study will provide valuable information on the effects of a KD on various aspects of health without the influence of obesity and without the confounding effects of weight loss. In particular, the results of this study will provide information on short-term dietary effects on sleep quality, cognitive function and measures of inflammation and cardiovascular health, but also on thyroid function and associated BMR.

### Trial status

Subject recruitment began in May 2017. We anticipate data collection to be complete by February 2018.

## Additional file

Additional file 1: SPIRIT 2013 fillable checklist indicating the position of recommended items in the manuscript. (DOC 121 kb)

## Abbreviations

BMI: Body Mass Index
BMR: Basal metabolic rate
BOHB: β-hydroxybutyrate
CRP: C-reactive protein
CVD: Cardiovascular disease
EAT: Exercise-associated thermogenesis
GHQ: General Health Questionnaire
GPAQ: Global Physical Activity Questionnaire
HCLF: High-carbohydrate, low-fat
HDL: High-density lipoprotein
IL-1: Interleukin-1
IL-6: Interleukin-6
KD: Ketogenic diet
LDL: Low-density lipoprotein
NEAT: Non-exercise-associated thermogenesis
NREE: Non-resting energy expenditure
PI: Principal investigator
PSQI: Pittsburgh Sleep Quality Index
RCTs: Randomized controlled trials
T3: Triiodothyronine
T4: Thyroxine
TEF: Thermic effect of food
TNF-α: Tumor necrosis factor alpha
TSH: Thyroid stimulating hormone
VAS: Visual Analogue Scale
